# Understanding COVID-19 Vaccine Hesitancy among the General Population in Japan from Public Health Ethical Perspectives: Findings from a Narrative Review

**DOI:** 10.1007/s41649-024-00310-8

**Published:** 2024-10-24

**Authors:** Moe Kuroda, Md Koushik Ahmed, Kaku Kuroda, Sandra D. Lane

**Affiliations:** 1 Norton College of Medicine, MPH Program, SUNY Upstate Medical University, Syracuse, NY USA; 2 Institute for Global Health and Translational Science, SUNY Upstate Medical University, Syracuse, NY USA; 3 Department of General Medicine, University of Toyama Hospital, Toyama, Japan; 4 Department of Public Health, Falk College of Sports and Human Dynamics, Syracuse University, Syracuse, NY USA; 5 Division of Geriatrics & Aging, Department of Medicine, University of Rochester, Rochester, NY USA; 6 Department of Obstetrics and Gynecology, SUNY Upstate Medical University, Syracuse, NY USA

**Keywords:** COVID-19, COVID-19 vaccine, Vaccine hesitancy, Vaccine acceptance, Japan

## Abstract

Japan has been reported as a country with high levels of vaccine hesitancy. However, a lack of comprehensive reviews studying factors for vaccine hesitancy for the COVID-19 vaccines in the Japanese context from the perspective of ethical controversy exists. Using a narrative review method, we reviewed factors associated with vaccine hesitancy to the COVID-19 vaccines and examined issues related to ethical controversy among the Japanese population. Factors associated with vaccine hesitancy include concerns about vaccine safety, suspicion of vaccine inefficacy, mistrust of the government, and low perceived threat. Factors associated with vaccine acceptance include environmental factors, factors related to Japanese cultural values, including collectivism and social norms, and positive attitudes toward information provided by authorities. Unique backgrounds in Japan are historical events such as the anti-HPV vaccine campaigns, the accessible medical system fostering high expectations of zero risk, and cultural factors of caring social norms influencing vaccine acceptance. Ethical controversies arise from preferences and practices at the individual or national level around individual rights versus public health benefits. Healthcare professionals and public health experts should continue dialoguing with the critical mass, practitioners, and policymakers, considering the ethical dilemmas surrounding individual rights and public health benefits. Insights obtained from this study indicate the need to develop tailored strategies to enhance vaccine acceptance while respecting individual autonomy within the Japanese context.

## Introduction

### History of Vaccine Hesitancy in Japan

Vaccine hesitancy, which has been defined as “the reluctance or refusal to vaccinate despite the availability of vaccines” by the World Health Organization (WHO), was listed as one of the ten threats to global health by WHO in 2019 (WHO 2019). Japan has been well-documented for its vaccine hesitancy, as represented by the delayed introduction of multiple vaccinations to the national immunization program compared to other high-income countries (Saitoh and Okabe 2012; de Figueiredo et al. 2020; Wong et al. 2021).

Several historical adverse events and the subsequent attitude of the government, published in mass media, have been reported to affect vaccine hesitancy in Japan (Nakano 2023; Saitoh and Okabe 2012). Initially, the national immunization program implemented in 1948 prevailed, contributing to decreasing vaccine-preventable diseases, such as pertussis, measles, etc. (Nakayama 2013; Saitoh and Okabe 2012). However, there were two deaths after the diphtheria, tetanus-toxoid, and whole cell pertussis (DTwP) vaccine in 1975 (Saitoh and Okabe 2012; Nakayama 2013). Even though the vaccine distribution was resumed after temporary pausing, the vaccination rate dropped (Saitoh 2014). Also, aseptic meningitis caused by the mumps component of mumps-measles-rubella (MMR) was reported in 1989 (Nakayama 2013; Saitoh and Okabe 2012). The mass media sensationally reported both events and patients' groups filed several lawsuits against vaccinations (Nakayama 2013; Saitoh and Okabe 2012). As a result, the Japanese government changed mandatory immunization programs to “effort-duty” vaccination programs in 1994, and other vaccines were assigned to “voluntary vaccinations” (Saitoh and Okabe 2012). Consequently, the government became reluctant to add new vaccines to the routine national immunization programs, and the development of domestic vaccines was delayed. For example, as of 2012, some significant vaccines administered in regular vaccines, such as the pneumococcal vaccine for adults and children, the Haemophilus influenzae type b (Hib) vaccine, the mumps vaccine, varicella zoster virus (VZV), the hepatitis B virus (HBV) vaccine, Measles and Rubella (MR) vaccine, Mumps vaccine, the Human Papilloma Virus (HPV) vaccine, Rotavirus vaccines in other high-income countries, were not assigned to a routine but set to a voluntary vaccine in Japan (Saitoh and Okabe 2012; Nakano 2023). As a result, Japan experienced a resurgence of measles and rubella (Ujiie 2019; Shimizu et al. 2020; Kitano 2021). Additionally, the lack of governmental recommendation for the booster of pertussis vaccine in school-age and pregnant women is also a significant example of the negative attitude towards vaccination in Japan(Hiiragi et al. 2021; National Institute of Infectious Diseases, Center for Epidemiology of Infectious Diseases and Bacteria Division II 2022).

Another recent important event was the withdrawal of the active recommendation for HPV vaccines in 2013, just two months after the government started a proactive recommendation for HPV vaccines in the same year. Repeated media reports on multiple potential adverse events led the patient groups to sue the government. Even though there was no significant association between these symptoms and the HPV vaccine in 2014, the Japanese government suspended the proactive recommendation of the HPV vaccine (Hanley et al. 2015; Saitoh and Okabe 2014). As a result, data from Hokkaido in Japan showed that the HPV vaccine completion rate dropped from over 70% to 0.6% among eligible girls after the suspension (Hanley et al. 2015; Simms et al. 2020; Ujiie et al. 2022). Another study estimated 5000–5700 deaths due to potential cervical cancer among females born between 1994 and 2007 who missed vaccination, brought by a significant drop in HPV vaccine rate from over 70% to less than 1% (Simms et al. 2020).

With these historical backgrounds and context, it is crucial to document how the concept of “vaccine hesitancy” is understood among the general population and healthcare professionals and how it relates to ethical controversies in the context of Japanese COVID-19.

### COVID-19 Vaccine Hesitancy and Acceptance in Japan

During the COVID-19 pandemic, vaccine hesitancy became a more significant issue, as shown in Japan's delay in vaccine roll-out and distribution compared to other countries. The roll-out of the COVID-19 vaccines for healthcare professionals in Japan started on February 17, 2021 (Ministry of Health, Labour, and Welfare, Japan 2020). However, the United States (U.S.), Canada, and the United Kingdom (U.K.) emergently approved the COVID-19 vaccine. They started the vaccine roll-out in December 2020 (Assistant Secretary for Public Affairs 2022). As of May 2021, only 5.9% of the population had been vaccinated in at least one dose in Japan, whereas the vaccination rate was 56% in the UK, 51% in the U.S., and 49% in Canada (Mathieu et al. 2020).

However, the vaccination rate increased gradually as Japanese society experienced a drastic surge in the number of positive cases during the summer of 2021, and it reached 80% as of December 2021 (Mathieu et al. 2020), which was higher than the U.S. and UK at that point.

### Ethical Controversy of Vaccine Hesitancy

From the ethical perspective of public health, vaccine hesitancy implies a controversy between individual freedom of refusal to be vaccinated and the collective good of the population’s health achieved by herd immunity through vaccination (Giubilini 2021). Giubilini, et al. discussed three theories that counter-argued individual liberty rights of refusal: utilitarianism, contractualism, and the principle of duty of easy rescue (Giubilini et al. 2018). In the theory of utilitarianism, described as “greatest good for the greatest number,” maximizing group benefit, which is building herd immunity in terms of the discussion of vaccination, prioritizes society’s benefit over the individual’s refusal to be vaccinated, as long as the benefit of being vaccinated surpasses the negative cost of vaccination (Giubilini et al. 2018; Ochola 2023; Pavey et al. 2024). The theory of utilitarianism, which emphasizes an individual’s moral obligation to be vaccinated to maximize greater societal welfare and benefits, has been fundamental in the public health policy to widely distribute vaccinations, including coerced vaccine policies (Su et al. 2021; Ochola 2023). However, it is crucial to consider the ethical counter-argued concept, the right of individual autonomy, when utilitarianism is applied to public health policymaking (Ochola 2023). The second theory, contractualism, which addresses how people should act, is based on unwritten principles to which all could agree or would agree under certain ideal circumstances (Giubilini et al. 2018; Ashford and Mulgan 2018). Based on this approach, people have a moral obligation to be vaccinated only if vaccination takes a small cost to individuals (Giubilini et al. 2018; Pavey et al. 2024). Third, a principle of “duty of easy rescue” also previously reported to support the moral obligation to be vaccinated among the general public, including both adults and children (Giubilini et al. 2018; Savulescu et al. 2021). “The duty of easy rescue” claims “when I could do something that entails a small cost to me and a significant benefit to others, I have a moral duty to do it.” Giubilini et al. mentioned that one of the rescue duties is an uncontroversial and responsible approach to justify the collective moral responsibility to achieve herd immunity (Giubilini et al. 2018). On the other hand, the “cost” of rescue differs depending on the individual, such as age difference. For instance, vaccination might be an easy rescue for the older generation. However, it might not be for the younger generation, given their relatively lower risk of being severely affected by COVID-19 (Savulescu 2023; Cameron et al. 2021). Those arguments based on utilitarianism, contractualism, and the duty of easy rescue agree to request an individual's moral responsibility for vaccination in society (Giubilini et al. 2018).

As represented by utilitarianism, those theories can justify the vaccine distribution policy over individual rights to refuse, including vaccine allocation and vaccine mandatory policy (Giubilini et al. 2018; Savulescu et al. 2021). Nevertheless, policies that mandate vaccines contain the risk of making vaccine-hesitant people more defensive (Qunaibi et al. 2021; Lin et al. 2020). Therefore, promoting self-acceptance by providing information about the merits of vaccines and/or nudging towards vaccination is suggested, other than mandate policy by WHO and other experts (World Health Organization 2022; Nakada et al. 2022; Lazarus et al. 2021).

### Ethical Controversy and Japanese Context

Several previous articles argued that Japan’s Confucian cultural values have led to the frequent characterization of Japanese society toward less individualism and more social cohesion, in contrast to the U.S. and other Western countries’ greater emphasis on individualism (Uehara et al. 2022; Liu 2018; Tucker 2022). Confucian philosophy emphasizes collectivism (Duan et al. 2022), as in the emphasis on group goals to individual goals and values. In such a context, people might tend to follow theories focusing on the public good. On the other hand, younger generations in Japan are reported to have a greater focus on individualism (Yoshizaki 1997). This tension between respect for traditions and the contemporary questioning of authority on the part of younger individuals might have also reflected the issue of vaccine hesitancy. Thus, the cohesive Japanese culture that emphasizes collective social norms might have changed somewhat, which could have affected the attitudes toward vaccine uptake among Japanese citizens.

### Research Gap and Aim

Although studies investigating COVID-19 vaccine hesitancy and the transition to a high vaccination rate exist in Japan, there are no comprehensive reviews studying factors for vaccine hesitancy in the Japanese context of healthcare system and cultural backgrounds from the perspective of ethical controversy, which mainly indicates the conflict between individual autonomy and collective good represented by Confucianism, that is still described by the scholars remaining the strong presence in Japanese values.

The present study aims to investigate factors associated with vaccine hesitancy to the COVID-19 vaccines among the Japanese population, explore researchers’ perspectives on those factors, and discuss the ethical controversy about COVID-19 vaccine hesitancy in Japan using the narrative review method.

## Methods

### Research Design and Researcher’s Background

A narrative review is traditionally called a “non-systematic review,” which allows researchers to identify broader research questions, include various studies, and provide a comprehensive summary, with interpretation and critique from different or unusual viewpoints (Gregory and Denniss 2018; Sukhera 2022). Exploring historical perspectives of the chosen topic and development of a problem or its management are good examples of what narrative review can cover (Gregory and Denniss 2018; Green et al. 2006; Sukhera 2022; Ferrari 2015).

The authors selected a narrative review due to the characteristics of the present study’s research questions elucidating factors associated with COVID-19 vaccine hesitancy in Japan and experts’ views about those factors, and the following ethical discussion on the results. For the first research question, to elaborate on what factors have affected vaccine hesitancy for the COVID-19 vaccine in Japan despite the importance of achieving herd immunity by high prevalence of immunization, authors needed to select, analyze and interpret articles using the chronological background of each article (Ferrari 2015). The second research question is to explore experts’ perspectives on the ethical controversy of vaccine hesitancy and acceptance of the COVID-19 vaccine in Japan. The characteristics of the research question, which is broad and not a specific clinical question, fit the design of the narrative review (Green et al. 2006).

Based on a narrative review design, this review followed the narrative methodological framework, which other researchers have widely used (Green et al. 2006; Ferrari 2015; Baethge et al. 2019; Kuipers et al. 2022). According to the narrative review’s framework, we conducted a preliminary literature search, synthesized available literature, and reported the results according to our research questions.

Research questions were determined based on the literature review and the author’s background. The first author and the third author (MK and KK) are Japanese family physicians who were studying public health and public health ethics in New York state of the U.S., amid the COVID-19 pandemic, which allowed them to critically review the literature, come up with this research questions, and focus on this ethical controversy. KK is also a family physician in the U.S.. Therefore, the research questions were both dependent on the review of the literature and knowing the Japanese medical system and living and working in the U.S.. The second author (MKA), who is another main author, is a public health professional with experience writing a review article. The last author (SDL) is an anthropologist and public health professional.

### Literature Search

We conducted literature searches in three databases, including PubMed, Scopus, and CINAHL. We considered articles published in English. In addition, we also used Google Scholar for articles in the Japanese language. We considered the articles that were published from January 1, 2020, to December 31, 2022. Search terms were chosen to address the following research question: “What factors have been affected COVID-19 vaccine hesitancy in Japan?”. The search strategy development included a brief review of the studies related to COVID-19 vaccine hesitancy done in other countries and Japan. Search terms are shown in Table 1.

**Table 1:** Search terms and strings

Search engine		Key terms
English peer-reviewed articles	PubMed	((("COVID-19" OR "coronavirus" OR "SARS-CoV-2") AND (Vaccine OR Vaccination)) AND (hesitancy OR refusal OR fear OR unwilling)) AND Japan
	Scopus	((("COVID-19" OR "coronavirus" OR "SARS-CoV-2") AND (Vaccine OR Vaccination)) AND (hesitancy OR refusal OR fear OR unwilling)) AND Japan
	CINAHL (EBISCO)	((("COVID-19" OR "coronavirus" OR "SARS-CoV-2") AND (Vaccine OR Vaccination)) AND (hesitancy OR refusal OR fear OR unwilling)) AND Japan
Japanese peer-reviewed articles	Google Scholar	((("COVID-19" OR "新型コロナウィルス" OR "SARS-CoV-2") AND "ワクチン" ) AND "忌避 ") AND "日本"

### Selection of Literature

We used the following inclusion and exclusion criteria (Table 2) for the final selection of studies to explore the present study’s research questions to identify associated factors and experts’ opinions about the COVID-19 vaccine hesitancy in Japan and to provide ethical discussion based on the review results. We included original articles / review articles published in peer-reviewed journals to delve into the views of experts stemming from the results quantitatively/qualitatively analyzed about the COVID-19 vaccine hesitancy in Japan critically reviewed (Green et al. 2006). The selection of studies included a two-stage selection process. At first, titles and abstracts were screened. Secondly, a full-text screening of potentially selected studies was conducted. Full-text screening in English was done independently by two reviewers (MK and MKA), and full-text screening in Japanese was done independently by two reviewers (MK and KK). Articles were considered eligible if they focused on or studied COVID-19 vaccine hesitancy in the Japanese context. The articles excluded by one reviewer were checked by another reviewer. Four authors (MK, MKA, KK, and SDL) discussed the disagreements and reached an agreement. Two authors (MK and KK) are native Japanese speakers. Each inclusion and exclusion was finalized upon detailed discussion and analysis based on the criteria.

**Table 2:** Inclusion and exclusion criteria for the selection of studies

Inclusion criteria	Exclusion criteria
• Papers focusing on the COVID-19 vaccine acceptance/hesitancy • Papers focusing on or using data from Japan • Published in English or Japanese • Published from January 1st, 2020 to December 31st, 2022, and • Original article and Narrative review and Review articles	• Vaccine hesitancy does not involve COVID-19 Study protocol without finding • Outcome does not involve COVID-19 vaccine uptake or intention, and • Study protocol, Letter to the editor, Editorial, or Comments

### Review of the Studies

After the final selection, we conducted a full-text review using a priori structured matrix. The priori structured matrix was developed by MK, reviewed by MKA, and supervised and finalized by SDL. The matrix included information from studies on objectives, study approach, study design, data analysis, study period, study population, sample size, and critical findings. After extracting data from all studies, the second reviewer (MKA) checked the data accuracy.

### Data Collection

**Fig. 1:**
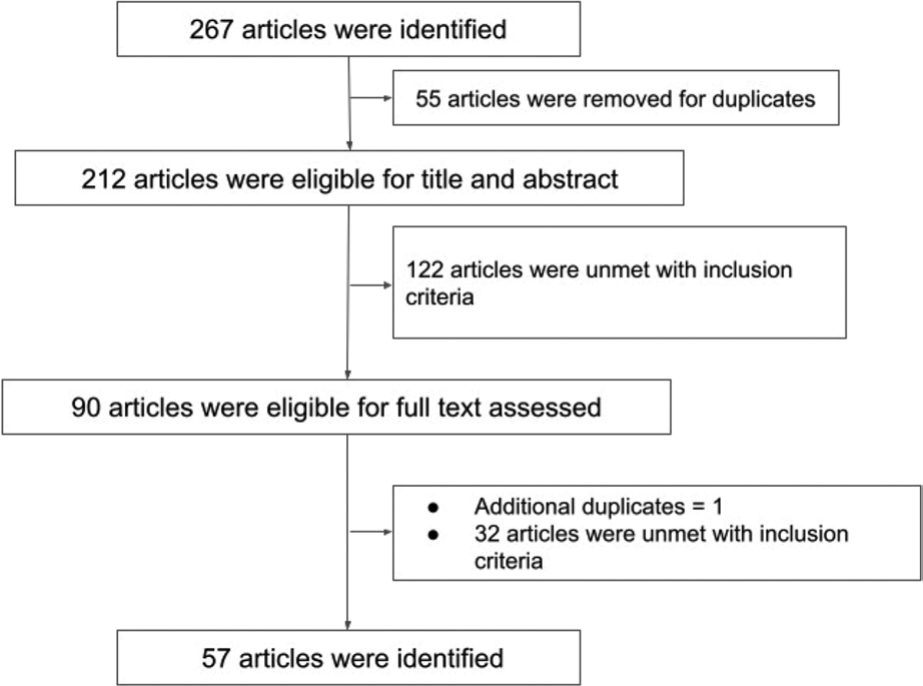
Flow diagram of the article screening process

Through the search in three databases, 267 articles were identified. After removing duplicates, 212 articles were retained for title and abstract screening. 55 articles were unmet with inclusion criteria. After removing one additional duplicate and 33 articles that did not meet the inclusion criteria, 57 articles were finally included in the content analysis. All processes are shown in Fig. 1.

### Analysis

We used descriptive statistics such as frequency counts and percentages to give an overview of the studies. Secondly, we used a thematic content analysis approach to critically analyze the factors associated with COVID-19 vaccine hesitancy.

## Results

### Study Characteristics

A total of 57 studies explaining vaccine hesitancy in Japan about the COVID-19 vaccines were explored to be analyzed (Table 3). Even though inclusion criteria included the publication year from 2020 to 2022, no studies were published in 2020. Most studies are primary research articles (*n* = 54/57; 94,7%). The most prevalent study design was cross-sectional (*n* = 44/57; 77.2%). In terms of the study population, various populations were identified, although most studies targeted the general population (*n* = 24/57; 42.1%), including two articles conducted in specific areas where the emergency declaration was announced at that time. There were articles targeted to specific age groups, such as the young generation (*n* = 6/57; 10.5%) and the working-age population (*n* = 1/57; 1.8%). 3 articles targeted workers (*n* = 3/57; 5.3%). Some articles targeted the healthcare field, including healthcare professionals (*n* = 4/57; 7.0%) and medical students (*n* = 2/57; 3.5%). Some articles aimed to understand the parental/pregnant/fertile generation, including articles targeting parents (*n* = 2/57; 3.5%) and articles targeting pregnant women (*n* = 3/57; 5.3%). Patients of the specific were also targeted (*n* = 2/57; 3.5%). One article targeted the migrant population (n = 1/57; 1.8%). Some articles analyzed the quotes in social media (*n* = 2/57; 3.5%) and social media users (*n* = 1/57; 1.8%).

**Table 3:** Characteristics of included studies (*n* = 57)

Characteristics	*N*	(%)
Year of publication		
2020	0	0
2021	39	68.4
2022	18	31.6
Study type		
Primary research	54	94.7
Review	3	5.3
Study approach		
Quantitative		
Cross-sectional study	43	75.4
Cross-sectional study, longitudinal study, and conjoint experimental design	1	1.8
Randomized conjoint study	1	1.8
Longitudinal study	2	3.5
Retrospective cohort study	3	5.3
Prospective cohort study	1	1.8
Before-and-after trial	1	1.8
RCT	1	1.8
Qualitative	1	1.8
Review	3	5.3
Study population		
General population	24	42.1
People who completed all doses of the COVID-19 vaccine	1	1.8
Young generation	6	10.5
Healthcare professionals	4	7.0
Medical students	2	3.5
Workers	4	7.0
Working age (64 years old or younger)	1	1.8
Parents	2	3.5
Pregnant women	3	5.3
Specific disease	2	3.5
Migrant population	1	1.8
Users of social media or apps	1	1.8
Quotes from social media	2	3.5
Review, RCT	4	7.0

*RCT* Randomized controlled trial

### Sociodemographic Factors associated with COVID-19 Vaccine Hesitancy in Japan in 2021–2022

Various factors associated with vaccine hesitancy were reported targeting the general population. We listed sociodemographic factors below, which have a statistically significant association with vaccine hesitancy reported in the manuscript. Factors were identified from the main text of the articles.

Major demographic factors reported were, younger age (Yoda and Katsuyama 2021; Machida et al. 2021a; Okamoto et al. 2022a; Kadoya et al. 2021; Nomura et al. 2021; Kobayashi et al. 2022; Suzuki et al. 2022; Arashiro et al. 2022; Odani et al. 2022; Tokiya et al. 2022a; Yoda et al. 2022; Yoneoka et al. 2022), female gender (Machida et al. 2021a; Okubo et al. 2021a; Okamoto et al. 2022a; Kadoya et al. 2021; Nomura et al. 2021; Kobayashi et al. 2022; Suzuki et al. 2022; Odani et al. 2022; Yoda et al. 2022), low-income level (Machida et al. 2021a; Okubo et al. 2021a; Kadoya et al. 2021; Nomura et al. 2021), low household assets (Kadoya et al. 2021), low financial literacy (Kadoya et al. 2021), non-full-time working or unemployed (Odani et al. 2022), being pregnant (Odani et al. 2022), married marital status (Okubo et al. 2021a), living alone (Okubo et al. 2021a), unmarried marital status (Ghaznavi et al. 2022; Suzuki et al. 2022), occupation outside the health care profession(Okubo et al. 2021a; Suzuki et al. 2022), occupation outside essential work in the food industry (Okubo et al. 2021a), low educational level (Okubo et al. 2021a; Kadoya et al. 2021; Tanaka et al. 2023; Yoda et al. 2022), having no children (Kadoya et al. 2021; Suzuki et al. 2022), and current alcohol use (Okubo et al. 2021a).

Health-related demographics reported were self-reported poor health status (Ghaznavi et al. 2022), no presence of chronic condition (Suzuki et al. 2022), presence of comorbidities (diabetes mellitus, psychiatric disorder) (Okubo et al. 2021a)*,* presence of severe psychological distress (Okubo et al. 2021a), no history of psychological illness (Suzuki et al. 2022), non-yearly influenza vaccine history (Ghaznavi et al. 2022), COVID-19 infection/testing history (Ghaznavi et al. 2022), lack of engagement in COVID-19 preventive measures (Ghaznavi et al. 2022), absence of underlying diseases or condition (Nomura et al. 2021; Yoda et al. 2022), history of a previous vaccine side effect (Kobayashi et al. 2022), and less concern about health status (Kadoya et al. 2021).

Characteristics of information sources associated with vaccine hesitancy reported were using specific information sources (internet news sites, YouTube, TikTok, Facebook, family members, and scientists and researchers (Nomura et al. 2021; Ghaznavi et al. 2022), trust in television (Nomura et al. 2021), and trust in magazines (Ghaznavi et al. 2022).

### Factors for COVID-19 Vaccine Hesitancy and Acceptance in Japan

Four themes of vaccine hesitancy and three themes of vaccine acceptance were identified as the reasons through thematic analysis of the narrative review (Table 4).

**Table 4:** Factors of COVID-19 vaccine hesitancy and acceptance in Japan extracted by thematic analysis

	Themes	Sub-themes
Vaccine hesitancy	Concerns about vaccine safety	Contextual skepticism for vaccine safety
		High Expectations for Medicine in Japan
		Misinformation
	Suspicion of vaccine inefficacy	
	Mistrust of government	
	Low perceived threat	Low susceptibility made by confidence in personal health
		Low perceived severity
		Lack of fear of getting infected
Vaccine acceptance	Environmental backgrounds	Gradual acceptance over time
		Increased access to vaccination
		Fear of infection overwhelming concerns of safety and efficacy
	Factors related to Japanese cultural values	Ethical motivation is driven by the responsibility to protect others
		Culture of keeping pace with others
		Fear of stigma when being infected
	Positive attitudes toward information provided by authorities	

### Factors of COVID-19 Vaccine Hesitancy in Japan

#### Theme 1. Concerns About Vaccine safety

##### Sub-Theme 1.1: Contextual Skepticism for Vaccine Safety

One of the important events related to contextual skepticism was the excessive emotional announcement by the media about the potential side effects after the HPV vaccine uptake among young females and the governmental decision of the suspension the regular occlusion of the HPV vaccine (Niu et al. 2022b; Saitoh et al. 2022; Yoda and Katsuyama 2021; Ishimaru et al. 2021; Kadoya et al. 2021; Nomura et al. 2021; Miyachi et al. 2022; Kajiwara et al. 2022; Yoda et al. 2022). Not only the HPV vaccine, the influenza vaccine (Yoda and Katsuyama 2021), and the MMR vaccine (Nomura et al. 2021) have also affected Japanese historical vaccine hesitancy, as is well known (Khan et al. 2022). The available evidence suggests that the memory of these events among the general public might have remained, contributing to an overarching skepticism toward general vaccine safety, even with the COVID-19 vaccines (Niu et al. 2022b; Saitoh et al. 2022; Yoda and Katsuyama 2021; Ishimaru et al. 2021; Kadoya et al. 2021; Nomura et al. 2021; Miyachi et al. 2022; Kajiwara et al. 2022; Yoda et al. 2022). Also, another factor related to contextual skepticism could be rooted in Japan's lack of primary care doctors. Japanese citizens have easy access to specialists and large hospitals instead of having primary care doctors. Moreover, the opportunities to visit clinics in person were limited amid the pandemic, which could make people have lower confidence in healthcare provider’s recommendations (Saitoh et al. 2022). This limited access to primary care physicians might hamper the provision of reliable, personalized information to assuage these safety concerns (Saitoh et al. 2022; Kimura et al. 2022).

##### Sub-Theme 1.2: High Expectations for Medicine in Japan

One study indicated the tendency of Japanese citizens to expect higher effectiveness and safety of the vaccine than the U.S., which could make people less tolerant to adverse events by the vaccines (Hara et al. 2021). Another study showed that Japanese adults prefer domestically developed vaccines and clinical trials for the COVID-19 vaccine compared to internationally developed vaccines and clinical trials (Kawata and Nakabayashi 2021).

##### Sub-Theme 1.3: Misinformation

Misinformation, especially through social media and TV, influenced concerns about vaccine safety (Miyachi et al. 2022; Okubo and Yoshioka 2021; Kimura et al. 2022; Tokiya et al. 2022a; Ferawati et al. 2022; Goodwin et al. 2022; Nomura et al. 2021; Yoneoka et al. 2022). False information such as “COVID-19 vaccine could damage reproductive function”, “the vaccine could make people infected with COVID-19” etc. have been reported (Goodwin et al. 2022; Harada and Watanabe 2021). Another example was the over-announcement about side effects in other countries. Overemphasized media reports about the side reactions in the U.K. and U.S. in December 2020, while the clinical trials had been still processed in Japan, might have affected vaccine hesitancy in Japan (Ishimaru et al. 2021). One of the characteristics of misinformation in Japan is that over-announcement of side effects by the media is more popular, while conspiracy is less popular (Takahashi et al. 2022a; Tokiya et al. 2022b). Misinformation has been widespread in Japan's anti-HPV and anti-MMR vaccine campaigns (Nomura et al. 2021).

#### Theme 2. Suspicion of Vaccine Inefficacy

The rapid development and approval of COVID-19 vaccines, while a remarkable scientific achievement, made some populations anxious about the efficacy in addition to the uncertainty of safety. These concerns, revolving around the perceived speed of development, have led some to question the thoroughness of the clinical trials and the long-term effects of the vaccines (Tanaka et al. 2023; Sugawara et al. 2021).

#### Theme 3. Mistrust of Government

Some studies mentioned a lower trust in the Japanese government's actions and decisions as an essential factor affecting vaccine hesitancy. Mistrust of the government could jeopardize both vaccine safety and efficacy (Hosokawa et al. 2022; Kobayashi et al. 2022; Goodwin et al. 2022; Suzuki et al. 2022; Nomura et al. 2021; Okubo and Yoshioka 2021; Tanaka et al. 2023). One study cited that the level of public trust in Japan's government is lower than in other countries (Kobayashi et al. 2022).

#### Theme 4. Low Perceived Threat

##### Sub-Theme 4.1: Low Susceptibility made by Confidence in Personal Health

Confidence in personal health diminished the susceptibility to COVID-19. This tendency is more popular among the young generation (Khan et al. 2021, 2022) or those with no particular medical histories (Suzuki et al. 2022). Consequently, these individuals perceive a lower urgency to vaccinate, considering themselves less susceptible to severe infection and to be contagious to others.

##### Sub-Theme 4.2: Low Perceived Severity

Low perceived severity was one of the essential psychological factors contributing vaccine hesitancy (Machida et al. 2021a, b; Miyachi et al. 2022; Okamoto et al. 2022a; Takahashi et al. 2022a). Low perceived severity can become a reason for vaccine hesitancy among the young generation, considering the older generation is at higher risk of becoming severely affected by COVID-19 infection, which makes the younger generation challenging to be willing to receive vaccines from the viewpoint of utilitarianism (Okamoto et al. 2022a).

##### Sub-Theme 4.3: Lack of Fear of Getting Infected

Also, a lack of concern (Okubo and Yoshioka 2021), and a lack of fear of getting infected or being infected by others, were reported to reduce the motivation toward vaccine uptake (Arashiro et al. 2022). Some studies reported that people with myopic views among the people were associated with vaccine hesitancy because those people don’t expect the future direction (Kadoya et al. 2021; Khan et al. 2021, 2022).

### Factors of COVID-19 Vaccine Acceptance in Japan

#### Theme 1. Environmental Backgrounds

##### Sub-Theme 1.1: Gradual Acceptance over Time

Acceptance came over time (Kadoya et al. 2021; Tokiya et al. 2022a; Hiraoka et al. 2022), as people gradually developed more trust and health literacy about safety and effectiveness as they see other’s experiences (Harada and Watanabe 2021); for example, the experiences of healthcare professionals (Tokiya et al. 2022a), friends (Hiraoka et al. 2022), families (Higuchi et al. 2022) and the media reports (Hiraoka et al. 2022; Kadoya et al. 2021) that the majority of people didn’t have severe side effects. Also, those who realized that the adverse reaction of the COVID-19 vaccine was not lethal recommended the COVID-19 vaccine to others. Especially, recommendations from crucial persons, such as family members (Higuchi et al. 2022), employers (Nomura et al. 2022), and doctors for pregnant women (Takahashi et al. 2022b), were reported. Thus, as time passed, the effectiveness and safety of the COVID-19 vaccine have been recognized, improving the vaccine acceptance rate among the general population (Nishida et al. 2021).

##### Sub-Theme 1.2: Increased Access to Vaccination

The establishment of large-scale vaccination sites by public agencies, universities, and large employers has improved accessibility for individuals to receive vaccines (Okubo et al. 2021b; Okamoto et al. 2022b; Mori et al. 2022; Tanaka et al. 2023). Additionally, opportunities for vaccination tied to employment, including job-related vaccination opportunities (Kadoya et al. 2021), have prompted the working population to receive vaccination.

##### Sub-Theme 1.3: Fear of Infection Overwhelming Concerns of Safety and Efficacy

People were more likely to get vaccinated when the threat of being infected tends to be lower than the concerns about vaccine safety and effectiveness (Niu et al. 2022a; Harada and Watanabe 2021). For example, the specter of infection due to a fourth wave of COVID-19 cases in the summer of 2021 in Japan has also driven many individuals to prioritize vaccination, motivated by the desire to avert a further rise in cases (Okubo et al. 2021b).

#### Theme 2. Factors Related to Japanese Cultural Values

##### Sub-Theme 2.1: Ethical Motivation Driven by the Responsibility to Protect Others

A prevalent ethical drive to protect close people, such as family members, or vulnerable populations of society, including older adults and immunocompromised, has engendered a sense of responsibility to contribute to collective immunity (Machida et al. 2021a; Koji et al. 2022).

##### Sub-Theme 2.2: Culture of Keeping Pace with Others

Several studies indicated that Japanese people tend to be influenced by the behavior and attitudes of others, such as friends, colleagues, community members, etc., which could affect high vaccine acceptance of the first and second doses in Japan (Hiraoka et al. 2022; Kimura et al. 2022). This tendency is expressed as putting the interest of society over the individuals (Okubo et al. 2021b), caring social norms (Tokiya et al. 2022b; Saitoh et al. 2022), herding effect (Mori et al. 2022), and groupism (Kajiwara et al. 2022). It is also negatively stated, such as “peer pressure” (Mori et al. 2022) or “subjective pressure” (Goodwin et al. 2022). One qualitative study also identified a fear of burdening family or friends when suffering from a severe condition due to COVID-19 among older adults as an example of the tendency to care for others in Japanese culture (Kimura et al. 2022). On the other hand, this tendency could also influence vaccine hesitancy (Kajiwara et al. 2022).

##### Sub-Theme 2.3: Fear of Stigma when being Infected

Being afraid of stigma or discrimination when people were infected affected their willingness toward vaccination, both among older adults (Yoda and Katsuyama 2021; Koji et al. 2022) and the young generation (Miyachi et al. 2022).

#### Theme 3. Positive Attitudes toward Information Provided by Authorities

Some studies indicated that trust in the information given by the government affected vaccine intention (Suzuki et al. 2022; Goodwin et al. 2022; Nomura et al. 2021). Also, one study reported that trust in information from the local authorities, medical professionals, and the government was associated with vaccine acceptance (Nomura et al. 2021). Furthermore, those who rely on physicians, nurses, television, and medical information sites as their source of information on COVID-19 are less likely to be unsure or unwilling to receive vaccines.

## Discussion

The ethical theory framework that we used in this paper highlights the tension and tradeoffs between individual autonomy and the common good. Our narrative review identified several key themes related to vaccine hesitancy, acceptance, and ethical controversy in the context of Japan, which is well-known as a country with one of the lowest vaccine confidence and trust in the world (de Figueiredo et al. 2020; Wong et al. 2021). Factors affecting vaccine hesitancy include concerns about vaccine safety, suspicion of vaccine inefficacy, mistrust of government, and low perceived threat. Also, for the factors influencing COVID-19 vaccine acceptance in Japan, environmental factors, factors related to Japanese cultural values, and individual ways of thinking were identified.

### COVID-19 Vaccine Hesitancy in Japan

This review revealed that concerns about vaccine safety and efficacy, mistrust of the government, and low susceptibility affected COVID-19 vaccine hesitancy in Japan. Two themes, concerns about vaccine safety, including three sub-themes: contextual skepticism, high expectations of medicine, misinformation, and mistrust of the government, have characteristics unique to Japan.

First, contextual skepticism and mistrust of the government were related to the history of the anti-HPV and anti-MMR vaccine campaigns and the passive attitudes of the government toward such movements, affecting distrust of vaccine safety among the general population in Japan. These findings were consistent with previous studies about the history of vaccine hesitancy in Japan (Nakano 2023; Saitoh and Okabe 2012, 2014). The following Japan-specific nature might influence this factor that these historical events have persistently affected vaccine hesitancy in Japan.

Second, high expectations for medicine also involve Japan-specific backgrounds as shown in several studies. In a cross-sectional study, it was reported that Japanese people do not tolerate being at risk of having adverse events by the vaccines (Hara et al. 2021). The medical system in Japan could cause it. Free access to medicine in Japan could make people believe that medicine should be perfect, making Japanese people less tolerant of side effects (Hara et al. 2021). Also, in a narrative review, it was said that “zero-risk,” which is a concept that “safety should be free from the risk,” is common in Japan (Nakamura 2021). Good access to medicine and Japanese society's “zero-risk” concept could affect COVID-19 vaccine hesitancy.

Third, misinformation in Japan also has different aspects from other countries regarding information content. While conspiracy belief is one of the major reasons for vaccine hesitancy in Western countries, conspiracy theories were less prevalent in Japan (Tokiya et al. 2022b; Hornsey et al. 2018; Takahashi et al. 2022a). Shibuya mentioned that COVID-19 vaccine hesitancy in Japan was not connected to the political argument and defensive attitude caused by the objection or anxiety by being violated of individual rights or freedom, which might affect less conspiracy theories related to the COVID-19 vaccine (Wingfield-Hayes 2021).

Based on the above, in light of the history of the anti-HPV and anti-MMR vaccine campaigns, it is imperative to call for appropriate information to the media and for promotion to fill in the gap between too-high expectations of zero risk and the potential for adverse events from vaccines.

### COVID-19 Vaccine Acceptance in Japan

This study found that environmental factors, psychological factors, and positive attitudes toward information provided by authorities impacted COVID-19 vaccine acceptance in Japan. Although environmental factors and positive attitudes toward information provided by the authorities were shown in previous studies targeting other countries (Reiter et al. 2020; Soares et al. 2021; Hill et al. 2021), some psychological factors were unique to Japan. The Japanese culture of keeping pace with others, which is also called “groupism” (Kajiwara et al. 2022) and “caring social norms” (Tokiya et al. 2022b; Saitoh et al. 2022), contributed to increasing vaccine acceptance. This concept puts importance on how others think and follow others’ behavior rather than the opinion of the individuals. The culture of “groupism” and “social norm” have high affinity with three principles arguing that the public health benefits to achieve herd immunity should be prioritized over individual rights, noted in utilitarianism, contractualism, and the principle of duty of easy rescue. Also, in Japanese collectivistic Confucian culture, vaccination is viewed as a prosocial activity prioritizing societal benefit over personal liberties (Uehara et al. 2022). One previous study pointed out that such a tendency, which could be considered “altruism,” is more prominent among people with more opportunities for social participation because they have a higher risk of infecting others (Komada et al. 2023). However, this concept could involve the possibility of the individual's right to freedom being violated by the public interest as a whole (Komada et al. 2023). Other expressions of this tendency that are negatively paraphrased, such as “peer pressure” (Mori et al. 2022) or”subjective pressure” (Goodwin et al. 2022), might alert this ethical controversy. At present, there is, especially among youth, rejection of the duty to put their autonomy on hold in favor of making their own decisions (Yoshizaki 1997). Thus, it is essential to take into account both the traditional solid "groupism" nature among the Japanese and the recent tendency to focus on individualism to promote new vaccines, monitoring if the approach is pressuring people to limit their autonomy.

### Limitation

The present study has some limitations. First, our research included only English and Japanese publications. However, we consider this approach suitable because the topic focuses on Japan, where both Japanese and English are used as primary languages for relevant literature. Also, our search was delimited to a specific database for literature search in Japanese. Notably, the Japanese articles database is widely acknowledged for its comprehensiveness, facilitating access to the most pertinent publications.

Second, we did not assess the quality of the included studies. Rather than excluding studies based on bias assessment, we focused on exploring researchers' perspectives and inclusivity by incorporating available and eligible studies and identifying emerging themes. These themes appropriately capture the authors' experiences and viewpoints.

## Conclusion

The present study identified important reported factors associated with COVID-19 vaccine hesitancy and acceptance, determined researchers’ perspectives on the COVID-19 vaccine hesitancy in Japan, and discussed the ethical controversy of those reasons unique to Japan. Historical events, including the suspension of the HPV vaccine, the accessible medical system, and the culture of groupism and caring social norms, were considered factors unique to Japan. This synthesis of factors associated with COVID-19 vaccine hesitancy and acceptance and how those relate to ethical controversies can help public health policymakers, government agencies, and researchers design better prevention strategies and best practices among the general population regarding adherence to public safety regulations. Although reasons for vaccine hesitancy should be addressed and reasons for vaccine acceptance should be more focused on for more effective vaccine distribution, it is essential to understand the ethical controversy between benefits for society and freedom of individual rights. Healthcare professionals and public health experts should continue to dialogue and discuss with the critical mass and policymakers, considering their ethical standpoints.

## Data Availability

The data of this study are available from the corresponding author upon reasonable request.
