# Correction to: Understanding COVID‑19 Vaccine Hesitancy among the General Population in Japan from Public Health Ethical Perspectives: Findings from a Narrative Review

**DOI:** 10.1007/s41649-024-00345-x

**Published:** 2024-12-04

**Authors:** Moe Kuroda, Md Koushik Ahmed, Kaku Kuroda, Sandra D. Lane

**Affiliations:** 1 Norton College of Medicine, MPH Program, SUNY Upstate Medical University, Syracuse, NY USA; 2 Institute for Global Health and Translational Science, SUNY Upstate Medical University, Syracuse, NY USA; 3 Department of General Medicine, University of Toyama Hospital, Toyama, Japan; 4 Department of Public Health, Falk College of Sports and Human Dynamics, Syracuse University, Syracuse, NY USA; 5 Division of Geriatrics & Aging, Department of Medicine, University of Rochester, Rochester, NY USA; 6 Department of Obstetrics and Gynecology, SUNY Upstate Medical University, Syracuse, NY USA

**Correction to: Asian Bioethics Review (2024)**

10.1007/s41649-024-00310-8

The article “Understanding COVID‑19 Vaccine Hesitancy among the General Population in Japan from Public Health Ethical Perspectives: Findings from a Narrative Review”, written by Moe Kuroda, Md Koushik Ahmed, Kaku Kuroda, and Sandra D. Lane, was originally published electronically on the publisher’s internet portal on 24 October 2024 without open access. With the author(s)’ decision to opt for Open Choice the copyright of the article changed on 28 November 2024 to © The Author(s) 2024 and the article is forthwith distributed under the terms of the Creative Commons Attribution 4.0 International License, which permits use, sharing, adaptation, distribution and reproduction in any medium or format, as long as you give appropriate credit to the original author(s) and the source, provide a link to the Creative Commons licence, and indicate if changes were made. The images or other third party material in this article are included in the article’s Creative Commons licence, unless indicated otherwise in a credit line to the material. If material is not included in the article’s Creative Commons licence and your intended use is not permitted by statutory regulation or exceeds the permitted use, you will need to obtain permission directly from the copyright holder. To view a copy of this licence, visit http://creativecommons.org/licenses/by/4.0.

The original article has been corrected.

